# FUT2 Secretor Status Is Not Associated With Oral Poliovirus Vaccine Immunogenicity in South Indian Infants

**DOI:** 10.1093/infdis/jiy553

**Published:** 2018-09-19

**Authors:** Edward P K Parker, Helena Whitfield, Chudarkodi Baskar, Sidhartha Giri, Jacob John, Nicholas C Grassly, Gagandeep Kang, Ira Praharaj

**Affiliations:** 1 Department of Infectious Disease Epidemiology, St Mary’s Campus, Imperial College London, United Kingdom; 2 Wellcome Trust Research Laboratory, Division of Gastrointestinal Sciences, Christian Medical College, Vellore, India

**Keywords:** Blood group, *FUT2*, immunogenicity, OPV, poliovirus, secretor, vaccine

## Abstract

*FUT2* determines whether histo-blood group antigens are secreted at mucosal surfaces. Secretor status influences susceptibility to enteric viruses, potentially including oral poliovirus vaccine (OPV). We performed a nested case–control study to determine the association between *FUT2* genotype (single-nucleotide polymorphisms G428A, C302T, and A385T) and seroconversion among Indian infants who received a single dose of monovalent type 3 OPV. Secretor prevalence was 75% (89 of 118) in infants who seroconverted and 80% (97 of 122) in infants who did not seroconvert (odds ratio, 0.79; 95% confidence interval, .43–1.45). Our findings suggest that *FUT2* genotype is not a key determinant of variation in OPV immunogenicity.

## Teaser

The secretion of histo-blood group antigens at mucosal surfaces is genetically determined (*FUT2*) and influences susceptibility to enteric viruses. However, based on a case–control study in India, we did not observe a significant association between *FUT2* genotype and oral poliovirus vaccine response.

The efficacy of oral vaccines in low-income countries is less than that in high-income countries. Several mechanisms have been implicated in this phenomenon, including interference by maternal antibodies, enteric pathogens, and environmental enteropathy [1]. Genetic factors may also be pertinent. In particular, the potential influence of histo-blood group antigen (HBGA) genotype on oral vaccines has garnered increasing interest.

HBGAs are glycans that are present on erythrocytes, on epithelial cells, and in mucosal secretions. The structure of these glycans is determined by several key loci. *FUT2*, for example, encodes a fucosyltransferase that modifies HBGAs such that they are secreted at mucosal surfaces—whereas individuals termed “secretors” have at least 1 functional copy of this gene, “nonsecretors” harbor mutations in *FUT2* that disrupt enzyme function. Notably, nonsecretors exhibit near-complete resistance to infection with P[8] rotavirus or GII.4 norovirus but remain susceptible to other genotypes of these enteric viruses [2], potentially reflecting genotype-specific HBGA binding.

Secretor status may also contribute to geographic discrepancies in oral vaccine efficacy. Although the frequency of secretors is generally comparable across populations in low- and high-income settings (approximately 80%), the implications of being a secretor may vary substantially. First, secretor status modifies susceptibility to enteric pathogens that display marked variation in transmission intensity. These enteric pathogens may, in turn, influence oral vaccine immunogenicity [3]. Second, the structure of secreted HBGAs depends on other genes (eg, *ABO* and *FUT3*), the alleles of which vary in frequency among populations [4]. Studies are starting to elucidate the complex relationship between HBGA phenotype and oral vaccine response. For example, among infants in Pakistan who received 3 doses of Rotarix, seroconversion was observed in 19% of nonsecretors, 30% of secretors with non-O blood groups, and 51% of secretors with blood group O [5]. In Nicaragua, rotavirus vaccine immunogenicity was reduced in infants with a Lewis A phenotype and was lower in secretors with blood group B than secretors with blood groups A or O [6].

The effect of secretor status on oral poliovirus vaccine (OPV) remains untested. We hypothesized that, analogous to the relationship observed for certain rotavirus and norovirus genotypes, nonsecretors may be less susceptible to replication of the poliovirus vaccine, inhibiting OPV immunogenicity. We tested this possibility by examining the association between *FUT2* genotype and vaccine response following a trial of monovalent type 3 OPV (mOPV3) in south India.

## METHODS

### Study Population

We recently performed a randomized, placebo-controlled trial to assess the impact of azithromycin on the immunogenicity of a single dose of mOPV3 among 754 infants aged 6–11 months in Vellore, India (CTRI/2014/05/004588). Details of the design and primary outcomes of the trial have previously been published [7]. All infants lacked type 3 poliovirus antibodies at enrollment. Blood samples were collected 21 days after vaccination to assess seroconversion, and any remaining blood was stored at 4°C. In a subset of 300 infants, poliovirus shedding was tested 7 days after vaccination. Written informed consent for enrollment was obtained from a parent or caregiver. The study was approved by the Institutional Review Board of the Christian Medical College and the Drugs Controller General of India.

Here, we report on a nested case–control study performed after the completion of this trial. We estimated that the inclusion of 123 serological responders and 123 nonresponders would provide 90% power with an α of .05 to detect a 2.5-fold increase in the odds of being a secretor among responders versus nonresponders, assuming an overall secretor genotype prevalence of 70% (based on a previous cohort study in Vellore [8]). We increased this sample size to 130 responders and 130 nonresponders to allow for an assay failure rate of 5% (eg, owing to insufficient DNA yield). Infants were considered eligible for inclusion if they completed the study per protocol, received placebo (as opposed to azithromycin), and had at least 500 μL of blood available for DNA extraction (n = 312). We prioritized infants for whom OPV shedding or bacterial microbiota composition had been assessed (as previously described [7, 9]) and selected the remaining infants at random (Supplementary Figure 1).

### FUT2 Genotyping by Real-Time Polymerase Chain Reaction (PCR) Analysis

DNA for *FUT2* genotyping was extracted from 500 μL of clotted blood, using the QIAamp DNA Blood Mini Kit. The clot was initially broken up with a pipette tip, then incubated with 500 μL of AL buffer and 20 μL of protease K for 30 minutes at 50°C. Before column-based purification, 400 μL of this mixture was subjected to a second incubation with 400 μL of AL buffer and 40 μL of protease K for 10 minutes at 56°C. Eluted DNA was stored at −70°C until testing.

In each DNA sample, we assessed 3 single-nucleotide polymorphisms (SNPs) that are known to disrupt the function of *FUT2*: G428A (rs601338), a nonsense mutation that is the most common cause of the nonsecretor phenotype globally; C302T (rs200157007), a missense mutation recently linked with secretor phenotype and enterotoxigenic *Escherichia coli* susceptibility in Bangladesh [10]; and A385T (rs1047781), a missense mutation that is prevalent in East Asians. The missense mutation G739A (rs602662) is also common globally but was found to co-occur with G428A in all South Asians in the 1000 Genomes Project [11, 12] and was therefore excluded here. Other polymorphisms have been shown to disrupt *FUT2* function but occur at low frequencies; for example, the missense mutation C571T (rs180028) has a minor allele frequency of 0.1% globally [12].

For SNPs G428A, C302T, and A385T, functional alleles are hereafter labeled Se^428^, Se^302^, and Se^385^, respectively, while nonfunctional alleles are labeled se^428^, se^302^, and se^385^. Individuals with 2 nonfunctional alleles at a given locus (eg, se^428^se^428^) will lack FUT2 activity, resulting in a nonsecretor phenotype. Moreover, owing to strong linkage disequilibrium between these SNPs, nonfunctional variants rarely co-occur on the same strand (full genotypic data from South Asians in the 1000 Genomes Project are provided in Supplementary Table 1). Thus, individuals heterozygous at >1 locus (eg, Se^428^se^428^ and Se^302^se^302^) will also lack a functional copy of *FUT2*.

SNPs were assessed on a QuantStudio 7 Flex real-time PCR system, using TaqMan SNP genotyping assays (Applied Biosystems; assay identifiers C__2405292_10, C_190470442_10, and C__8832449_10), following the manufacturer’s instructions. Genotypes were assigned using TaqMan Genotyper software (Applied Biosystems).

### Statistical Analysis

For each locus in turn, we assessed the association between secretor status (secretor vs nonsecretor as a categorical independent variable) and seroconversion via logistic regression. Infants were designated secretors if they had ≥1 functional allele (eg, Se^428^Se^428^ or Se^428^se^428^) and nonsecretors if they had 2 nonfunctional alleles (se^428^se^428^). We also performed a combined analysis in which infants were designated as nonsecretors if they had 2 nonfunctional alleles at any locus (se^428^se^428^, se^302^se^302^, or se^385^se^385^) or if they were heterozygous at >1 locus (eg, Se^428^se^428^ and Se^302^se^302^). In the subset of 109 infants for whom poliovirus shedding was tested 7 days after vaccination (of whom 67 [61%] were positive for poliovirus), we repeated the analysis with shedding as the outcome variable. Geometric mean titers of serum neutralizing antibodies were compared between secretors and nonsecretors, using the Wilcoxon rank sum test, with values of 1/3 and 1/728 assigned to infants with values below and above the limits of the microneutralization dilution series (1/4 and 1/512, respectively). Analyses were performed in the programming language R.

## RESULTS

Among the 260 infants included in this study, we successfully genotyped SNPs G428A, C302T, and A385T in 240 (92.3%); no amplification was observed for ≥1 SNP in the remaining samples. At SNP G428A, we observed the genotypes Se^428^Se^428^, Se^428^se^428^, and se^428^se^428^ in 147 (61.3%), 56 (23.3%), and 37 (15.4%) infants, respectively. At SNP C302T, we observed the genotypes Se^302^Se^302^, Se^302^se^302^, and se^302^se^302^ in 194 infants (80.8%), 38 infants (15.8%), and 8 infants (3.3%), respectively. At SNP A385T, 239 of 240 infants (99.6%) had the genotype Se^385^Se^385^, and the remaining individual had the genotype Se^385^se^385^. Nine individuals (3.8%) were heterozygous at SNPs G428A and C302T and were designated nonsecretors.

We did not observe a significant association between *FUT2* genotype and seroconversion status (Table 1). Combining across loci, secretor genotypes were observed in 89 of 118 infants (75.4%) who seroconverted to type 3 poliovirus and 97 of 122 infants (79.5%) who failed to seroconvert (odds ratio [OR], 0.79; 95% confidence interval [CI], .43–1.45; logistic regression, *P* = .445). Geometric mean titers of serum neutralizing antibodies 21 days after immunization were higher in nonsecretors than secretors, although this difference was not significant (19.2 vs 15.2; Wilcoxon *P* = .195; Supplementary Figure 2). When considering postvaccination shedding as an outcome, we did not observe a significant difference in secretor genotype prevalence between shedders and nonshedders (Table 1).

**Table 1.** Frequency of *FUT2* Alleles and Inferred Phenotypes by Oral Poliovirus Vaccine (OPV) Response

SNP, Group	Seroconversion, No. (%)			Shedding, No. (%)		
	Seronegative (n = 122)	Seropositive (n = 118)	*P*	Nonshedder (n = 49)	Shedder (n = 60)	*P*
G428A						
Se^428^Se^428^ or Se^428^se^428^	107 (87.7)	96 (81.4)	.176	43 (87.8)	52 (86.7)	.866
se^428^se^428^	15 (12.3)	22 (18.6)		6 (12.2)	8 (13.3)	
A385T						
Se^385^Se^385^ or Se^385^se^385^	122 (100)	118 (100)	-	49 (100)	60 (100)	-
G302T						
Se^302^Se^302^ or Se^302^se^302^	116 (95.1)	116 (98.3)	.184	47 (95.9)	58 (96.7)	.837
se^302^se^302^	6 (4.9)	2 (1.7)		2 (4.1)	2 (3.3)	
Combined^a^						
Secretor	97 (79.5)	89 (75.4)	.449	39 (79.6)	47 (78.3)	.873
Nonsecretor	25 (20.5)	29 (24.6)		10 (20.4)	13 (21.7)	

*P* values were determined via logistic regression with OPV outcome as the dependent variable. SNPs were not successfully genotyped in 20 of 260 infants included in this study (12 serological responders and 8 nonresponders).

Abbreviation: SNP, single-nucleotide polymorphism.

^a^Infants with the genotypes se^428^se^428^ and se^302^se^302^ were inferred to be nonsecretors, as were those with heterozygous genotypes at >1 locus (eg, Se^428^se^428^ and Se^302^se^302^).

## DISCUSSION

The secretion of HBGAs, as determined by *FUT2*, affects susceptibility to several enteric viruses. Although recent findings suggest that nonsecretors may be less likely to respond to oral rotavirus vaccine [5, 6], the same does not appear to be true of OPV. Among infants in south India—a population that has consistently been linked with impaired response to oral vaccines—the proportion of secretors did not differ significantly according to seroconversion or shedding status following a single dose of mOPV3.

Notably, in the case of both rotavirus and norovirus, viral surface proteins are known to bind HBGAs in a genotype-specific manner [13, 14]. It is likely that these interactions form the basis of discrepancies in susceptibility between secretors and non-secretors. The extent to which polioviruses interact with HBGAs is unknown. However, poliovirus has been shown to bind lipopolysaccharide, and in vitro infectivity is enhanced by exposure to this bacterial surface molecule [15]. Thus, it appears that poliovirus have evolved to exploit microbiota-derived signals during colonization of the intestinal mucosa, and may simply be indifferent to the presence or absence of HBGAs in mucosal secretions.

Our study has several limitations. First, we lacked the necessary samples to directly assess whether HBGAs were present in mucosal secretions and have therefore inferred phenotype from genotype. However, previous studies have found SNPs G428A and C302T to be highly predictive of secretor phenotype [6, 10]. Second, HBGA-related genes other than *FUT2*, such as *ABO* and *FUT3* (the Lewis gene), may also be pertinent to OPV outcome but were not considered here. Finally, we based our sample size calculations on a previous cohort study in Vellore in which one third of individuals were nonsecretors [8]. By contrast, we observed nonsecretor genotypes in 23% of infants. The allele frequencies at SNPs G428A, C302T, and A385T in this study were consistent with those observed among South Asians in the 1000 Genomes Project [11, 12], and reasons for the discrepancy with previous findings from Vellore (including a significantly lower prevalence of the allele se^385^ in the present study) are unclear.

These caveats notwithstanding, our study is among the largest to assess the association between *FUT2* genotype and oral vaccine response. Our findings suggest that the locus may not be a key determinant of variation in OPV immunogenicity.

## Supplementary Data

Supplementary materials are available at *The Journal of Infectious Diseases* online. Consisting of data provided by the authors to benefit the reader, the posted materials are not copyedited and are the sole responsibility of the authors, so questions or comments should be addressed to the corresponding author.

## Supplementary Material

Supplementary Material

## Notes

***Acknowledgments.***  We are grateful to Johan Nordgren, for advising on the *FUT2* genotyping methods, and to Revathi R, for her assistance with the PCR assays.

***Financial support.*** This work was supported by the UK Medical Research Council and the UK Medical Research Council and the Bill and Melinda Gates Foundation (grant OPP1039135).

***Potential conflicts of interest.*** All authors: No reported conflicts of interest. All authors have submitted the ICMJE Form for Disclosure of Potential Conflicts of Interest. Conflicts that the editors consider relevant to the content of the manuscript have been disclosed.
