# Supplementary material for: FUT2 Secretor Status Is Not Associated With Oral Poliovirus Vaccine Immunogenicity in South Indian Infants
Source: J Infect Dis. 2018 Sep 19;219(4):578–81. doi: 10.1093/infdis/jiy553 (PMC6350944; doi:10.1093/infdis/jiy553)
Supplement: Supplementary Material [file jiy553_suppl_supplementary_material.pdf]

Supplementary Table 1. ***FUT2*** **polymorphisms among South Asians in the 1000 Genomes Project.**

Genotype	G428A (rs601338)	C302T (rs200157007)	A385T (rs1047781)	C571T (rs1800028)	G739A (rs602662)[a]
SeSe	262 (53.6)	336 (68.7)	481 (98.4)	489 (100)	262 (53.6)
Sese[b]	177 (36.2)	127 (26)	8 (1.6)	0 (0)	177 (36.2)
sese	50 (10.2)	26 (5.3)	0 (0)	0 (0)	50 (10.2)

Data are n (%). Frequencies were obtained from http://grch37.ensembl.org.

[a] Locus is in perfect linkage disequilibrium with G428A (i.e. all nonfunctional alleles occur on the same strand).

[b] 54 (11%) individuals are heterozygous at two of G428A, C302T, or A385T. In all cases, nonfunctional variants occur on separate strands, such that the individuals will lack a functional copy of *FUT2*.

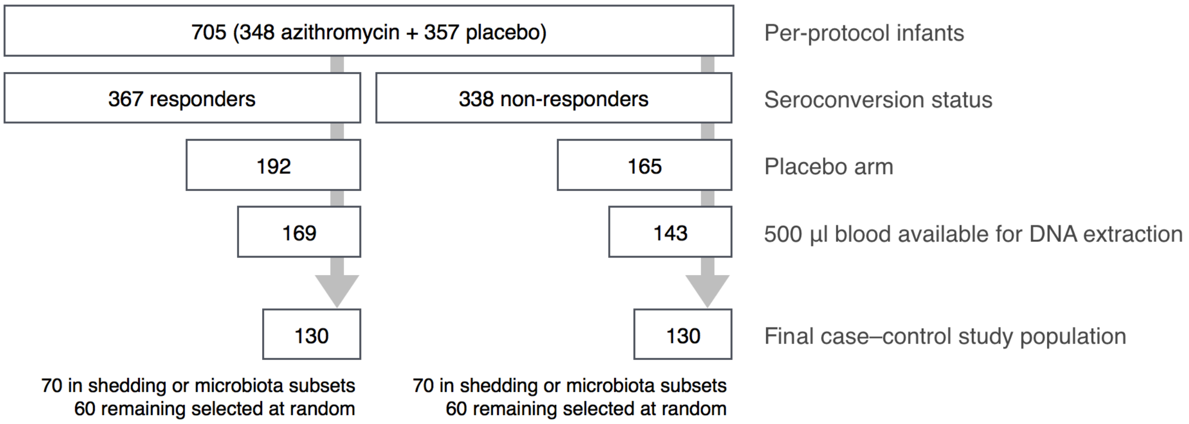

Supplementary Figure 1. Selection of study population. Oral poliovirus vaccine shedding was assessed in the first 300 infants with sufficient sample volumes for additional assays (including measurement of biomarkers of enteropathy) [7]. Microbiota composition was assessed in a randomly selected subset of the first 300 infants to be enrolled [9]. The shedding and microbiota subsets were selected independently.

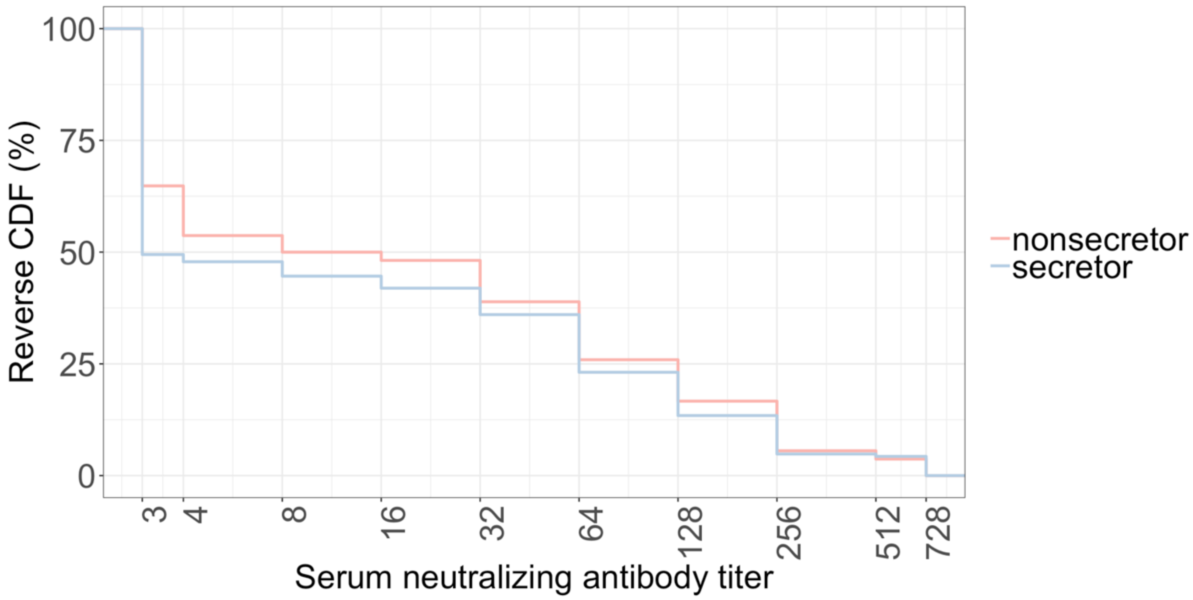

Supplementary Figure 2. Reverse cumulative distribution of postvaccination serum neutralizing antibody titers according to secretor status. Abbreviation: CDF, cumulative distribution function.
